# Use of Proteomic Imaging Coupled With Transcriptomic Analysis to Identify Biomolecules Responsive to Cochlear Injury

**DOI:** 10.3389/fnmol.2018.00243

**Published:** 2018-07-17

**Authors:** Kenyaria V. Noble, Michelle L. Reyzer, Jeremy L. Barth, Hayes McDonald, Michael Tuck, Kevin L. Schey, Edward L. Krug, Hainan Lang

**Affiliations:** ^1^Department of Pathology and Laboratory Medicine, Medical University of South Carolina, Charleston, SC, United States; ^2^Mass Spectrometry Research Center, Vanderbilt University, Nashville, TN, United States; ^3^Department of Regenerative Medicine and Cell Biology, Medical University of South Carolina, Charleston, SC, United States

**Keywords:** cochlea, hearing loss, MALDI-TOF IMS, microarray, noise, ouabain, non-sensory cells

## Abstract

Exposure to noise or ototoxic agents can result in degeneration of cells in the sensory epithelium and auditory nerve, as well as non-sensory cells of the cochlear lateral wall. However, the molecular mechanisms underlying this pathology remain unclear. The purpose of this study was to localize and identify proteins in the cochlea that are responsive to noise or ototoxic exposure using a complementary proteo-transcriptomic approach. MALDI imaging of cochlear sections revealed numerous protein signals with distinct cochlear localization patterns in both cochlear injury models, of which six were chosen for further investigation. A query of proteomic databases identified 709 candidates corresponding to m/z values for the six proteins. An evaluation of mRNA expression data from our previous studies of these injured models indicated that 208 of the candidates were affected in both injury models. Downstream validation analyses yielded proteins with confirmatory distributions and responses to injury. The combined analysis of MALDI imaging with gene expression data provides a new strategy to identify molecular regulators responsive to cochlear injury. This study demonstrates the applicability of MALDI imaging for investigating protein localization and abundance in frozen sections from animals modeling cochlear pathology.

## Introduction

Loss or dysfunction of the sensory hair cells, neural cells, and non-sensory cells in the cochlear lateral wall results in sensorineural hearing loss affecting millions of people worldwide. Exposure to noise causes degeneration of multiple cochlear cell types and structures, including the sensory hair cells, spiral ganglion neurons and synapses, and cochlear fibrocytes in the spiral ligament (Wang et al., 2002; Hirose and Liberman, 2003; Cui et al., 2011; Eskiizmir et al., 2011). Application of the ototoxic-reagent ouabain to the cochlea is a well-established model that selectively eliminates type I spiral ganglion neurons in the auditory nerve (Lang et al., 2005; Yuan et al., 2014). However, the molecular mechanisms underlying these pathological alterations remain unclear. Methods for global transcriptomic analysis, such as tissue microarrays and RNA sequencing, have been used to identify genes that are involved in responding to cochlear injury (Schuck et al., 2011; Yang et al., 2015). Studies using the aforementioned techniques have identified stress and immune response pathways as significantly affected by noise exposure in a rodent model (Patel et al., 2013; Yang et al., 2016).

However, one limitation to the study of animal models of sensorineural hearing loss is the small size of the cochlea, often requiring the pooling of tissues from many subjects for genetic and proteomic analysis. Quantification techniques that require whole cochlea and potentially pooled tissues are hampered by the fact that those techniques may miss changes occurring in spatial or cellular subdomains due to the heterogeneity of the tissue, e.g., the multiple cell types and ranged cell type distributions in structures such as the organ of Corti, auditory nerve, and cochlear lateral wall. Animal studies employing standard mass spectrometry have been able to identify unique protein components for cochlear cells and substructures under normal conditions (Yang et al., 2011). Mass spectral analysis also has been used to study pathological responses, for example, protein expression changes in response to the ototoxic drug cisplatin (Coling et al., 2007). Notably recent advances in understanding the localization of cisplatin accumulation in the cochlea were made using a form of imaging mass spectrometry (IMS) that detects metals (Brouwers et al., 2008; Breglio et al., 2017). However, these methods are not capable of observing protein distribution within cochlea tissue sections under normal or injury conditions. Such limitations are overcome by advances in matrix assisted laser desorption/ionization time-of-flight (MALDI TOF) IMS analysis, which allows detection of protein spatial localization within tissue sections and requires less sample manipulation than traditional mass spectrometry analysis.

MALDI-TOF IMS permits the identification of biomolecules within a thin tissue section while maintaining spatial information (Norris and Caprioli, 2013; Gessel et al., 2014). This procedure has been used successfully to map the distribution of lipids (Takizawa et al., 2011) and proteins (Hanrieder et al., 2012; Kakuda et al., 2017; Llombart et al., 2017) in tissue sections from various organs, including the brain and kidney. This imaging is accomplished by the uniform deposition of matrix across the surface of the tissue section, followed by laser desorption and ionization of the molecules in a raster fashion. Visualization of the acquired mass spectral data allows the localization and intensity information for any one mass-to-charge protein signal to be displayed across the entire section (Chaurand et al., 2005). This permits the identification of biological markers localized to specific tissue structures.

**Figure 1:**
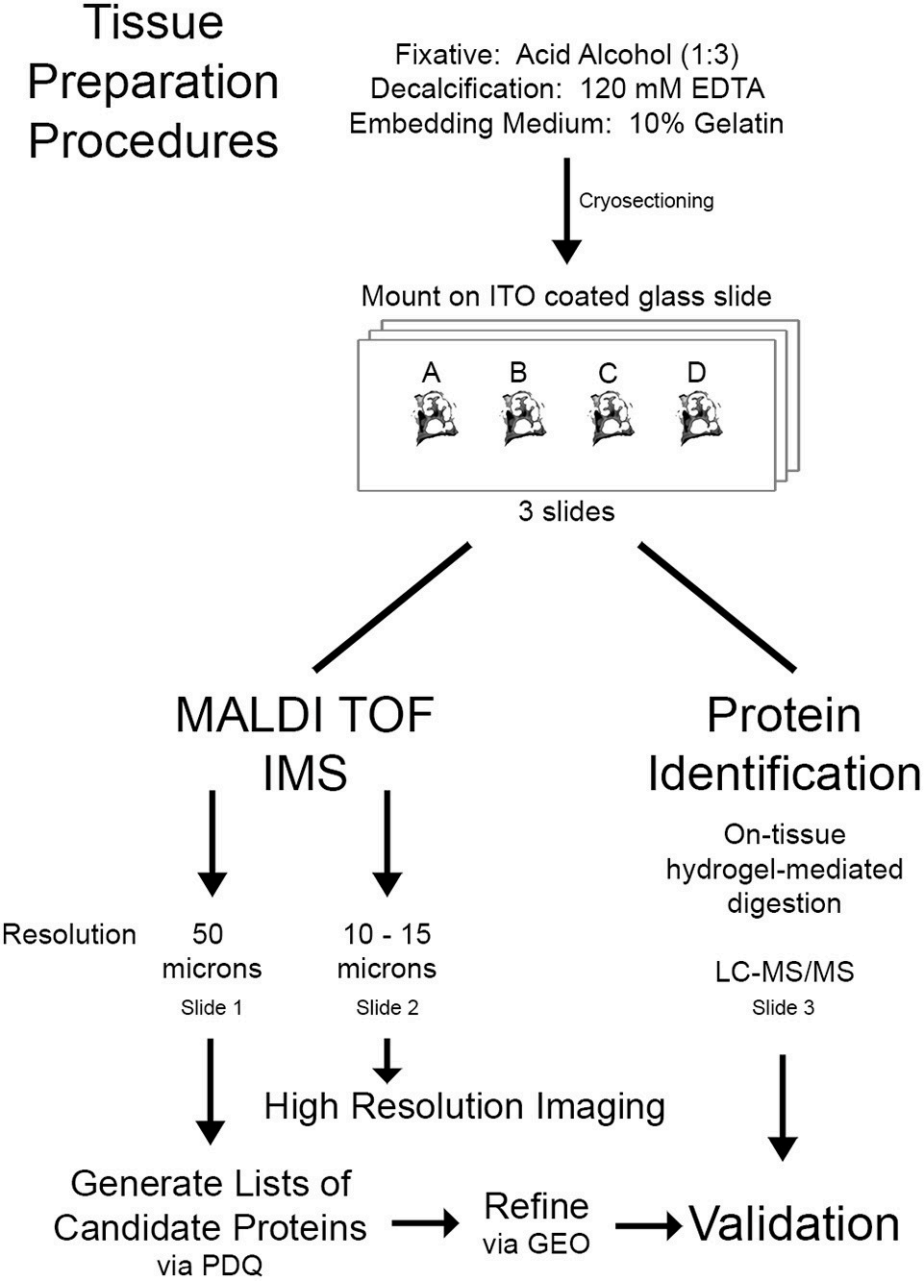
Workflow for proteomic imaging of the cochlea. Mouse cochleas were fixed in acid alcohol followed by decalcification and embedding in gelatin. A single 10 μm thick section from each condition [control (A), 106 dB 1 day (B), 112 dB 1 day (C), or ouabain 3 day (D)] was mounted on an ITO glass slide, totaling four sections per slide. A total of three slides with this layout were analyzed, two by IMS and one by LC-MS/MS. Slides were subjected to MALDI TOF IMS at 50 or 10–15 μm resolution or on-tissue hydrogel mediated digestion for protein identification. Lists of candidate proteins of interest were generated using protein database query (PDQ) and refined using our previous transcriptomic analysis of the auditory nerve samples obtained from ouabain- and noise-exposed mice (Lang et al., 2006, 2011) [datasets available at NCBI Gene Expression Omnibus (GEO)]. Validation was completed by comparing the list of candidate proteins to the proteins identified by LC-MS/MS.

The aim of this study was to apply MALDI TOF IMS to identify and acquire high-resolution spatial information for proteins responsive to cochlear insult. This allows the discrimination of protein changes in specific substructures of the cochlea. To achieve this, cochlear tissue sections from CBA/CaJ mice exposed either to noise or ouabain were subjected to MALDI-TOF IMS analysis (Figure 1). Numerous protein signals were found localized to the cochlear lateral wall and/or auditory nerve, and signal abundance (ion density) increased relative to the severity of insult. Lists of proteins were compiled by a query of proteomic databases and refined using mRNA expression data from our previous studies of these injury models (Lang et al., 2011; Panganiban et al., 2018). This was followed by validation of protein identity with liquid chromatography tandem MS/MS (LC-MS/MS). Our findings indicate that this methodology is conducive for the discovery of proteins responsive to cochlear injury.

## Materials and methods

### Animals

CBA/CaJ mice (Jackson Laboratory, Bar Harbor, ME) were housed and bred in the low noise animal research facility of the Medical University of South Carolina (MUSC). Mice were maintained on a 12 h light/dark cycle and provided food and water *ad libitum*. Four mice, 1 female and 3 males, aged 2 months, were used in the study. Animals were randomly assigned to control and experimental treatments with the result that the control animal was female and the three injury animals (106 dB noise, 112 dB noise, and ouabain exposure) were male. Subjects were evaluated to ensure that there was no evidence of (1) external ear canal obstruction, (2) middle ear obstruction, or (3) middle ear infection. All procedures outlined in this study were conducted in accordance with the guidelines of the Institutional Animal Care and Use Committee of MUSC.

### Auditory function measurement

Auditory brainstem response (ABR) was measured before and after insult as previously described (Lang et al., 2011). Briefly, anesthesia (20 mg/kg xylazine and 100 mg/kg ketamine solution) was administered by intraperitoneal injection with body temperature maintained at 37°C using a Delta Phase isothermal pad (DP-SASB, Braintree Scientific, Inc.). ABR tests were performed in a sound isolation booth using Tucker Davis Technologies System III equipment (Tucker Davis Technologies, Gainesville, FL) that was professionally calibrated before use. Sound stimuli were delivered into the ear canal via a 9.5 cm long, 3 mm diameter tube and responses processed via SigGen software package (Version 4.4.1). ABRs were evoked at frequencies 4–45.2 kHz with 5 ms tone pips with cos^2^ rise/fall times of 0.5 ms delivered 31 times/s. Sound levels were reduced by 5 dB steps, from 90 to 10 dB SPL. Pre- and post-insult responses for each subject were assessed for threshold at each frequency and plotted using Origin 6.0 software (OriginLab Corporation, Northampton, MA).

### Mouse model of noise-induced hearing loss

Modifications were made to the noise exposure protocol used in previous studies from our lab (Lang et al., 2006). Briefly, subjects were exposed to 8-16 kHz octave-band noise at 106 dB (*n* = 1) or 112 dB (*n* = 1) for 2 h using a Beyer DT48 drive (Beyerdynamic, Farmingdale, NY) and monitored with a probe-tube microphone (B&K 4134; Bruel and Kjaer, Norcross, GA). ABR tests and cochlear tissue collection were performed at 1 day post-noise exposure.

### Mouse model of selective SGN degeneration

Survival surgical procedures for ouabain exposure to the cochlea were performed under sterile conditions as previously described (Lang et al., 2011; Stevens et al., 2015). Briefly, the subject received intraperitoneal injection of buprenorphrine (0.1 mg/kg) 30 min before surgery to reduce discomfort during the procedure. Immediately before the procedure xylazine (20 mg/kg) and ketamine (100 mg/kg) were used for anesthesia. The postauricular approach was used to perforate the bulla following which 10 μl of 3mM ouabain (O3125, Sigma-Aldrich) was applied to the exposed round window niche. Every 10 min the volume was wicked away and a fresh aliquot applied for a total exposure time of 1 h. The operation was performed on the right ear and the left ear was the surgical control. After surgery, subjects were provided soft food and monitored closely over 1–3 days; ABR tests and tissue was collected 3 days after ouabain exposure.

### MALDI-TOF IMS

#### Sample preparation

Cochleas from one representative subject for each condition were prepared for MALDI-TOF IMS: control (female), 106 dB (male), 112 dB (male), and ouabain (male). Following end-point ABR measurement, mouse temporal bones were obtained and immersed immediately in a 1:3 (v:v) solution of acetic acid (Sigma Aldrich) and 200 proof ethanol (Decon Laboratories). The cochleas were dissected out in fixative, then perfused through the oval and round windows. Isolated cochlear tissues remained in the fixative overnight at −20°C and were then decalcified with 120 mM EDTA for 48 h and embedded in 10% gelatin for cryosectioning at 10 μm thickness. Modular-sections were mounted on indium-tin oxide (ITO) coated glass slides (Delta Technologies) and stored at −70°C in a slide cassette.

Frozen sections of cochlea tissues were allowed to come to room temperature prior to exposure to ambient air in order to minimize water condensation onto the samples. Once at room temperature, the sections were washed as follows: 70% ethanol, 30 s; 100% ethanol, 30 s; Carnoy's fluid (ethanol/chloroform/acetic acid, 6:3:1), 2 min; 100% ethanol, 30 s; water, 30 s; and 100% ethanol, 30 s. After washing, the slide was allowed to dry at room temperature prior to matrix coating.

#### Protein imaging

MALDI matrix 2,5-dihydroxyacetophenone (DHA) was spray-coated onto the MALDI target slide via an automatic sprayer (TM Sprayer, HTX Technologies, Chapel Hill, NC). DHA was made up at 15 mg/ml in 90% acetonitrile with 0.2% trifluoroacetic acid. Six passes were applied in a criss-cross spray pattern with a nozzle temperature of 85°C, a flow rate of 0.2 ml/min, 2 mm track spacing, and a stage velocity of 1,100 mm/min. The sections were rehydrated prior to analysis by warming the slide for 2 min at 37°C followed by exposure of the slide to 1 ml 50 mM acetic acid for 3 min at 37°C. Images were acquired with a MALDI TOF mass spectrometer (Rapiflex Tissuetyper, Bruker, Billerica, MA) equipped with a Smartbeam 3D 10 kHz Nd:YAG laser that was frequency tripled to 355 nm wavelength. Data were collected in the positive ion mode with the laser operating at 10 kHz. Two slides were used during the imaging analysis—the first slide for imaging of the entire tissue section for each condition (control, 106 dB, 112 dB, and ouabain) and the second slide for high resolution imaging. The pixel spacing was 10–50 μm (center-to-center distance) in both x and y dimensions for analysis of control and noise sections, and the ouabain treated sample was analyzed at 15–50 μm pixel spacing. Data were collected from mass-to-charge ratio (m/z) 2,000–20,000. Resulting data was visualized with flexImaging software (Bruker, Billerica, MA). Cochlear regions were identified by hematoxylin and eosin staining of sections post imaging.

### Protein identification via tagident tool

The TagIdent tool available on ExPASy (Wilkins et al., 1999) was used to generate lists of possible proteins for the observed m/z signals of interest. The m/z was used to define the molecular weight region as MALDI-generated ions are typically assumed to be singly charged. The molecular weight range for the analysis was defined as ± 1 kDa to account for mass error and potential modifications. The Swiss-Prot OS/OC (species/classification) was specified as *Mus musculus* corresponding to the TaxID 10090.

### Analysis of gene microarray data

Microarray data corresponding to 106 dB noise and ouabain exposure used in this study have been reported previously (Lang et al., 2011; Panganiban et al., 2018) and are deposited in NCBI Gene Expression Omnibus (accessions GSE100365 and GSE59416, respectively). Expression values normalized by RMA algorithm were used for comparative analysis. Genes significantly differentially expressed in the auditory nerve by either the ouabain treatment or the noise injury were defined as those having *p* < 0.05 (Student's *t*-test, unpaired, 2-tailed) for comparisons involving the experimental vs. control conditions. False discovery rate, estimated based on iterative comparisons using permuted group assignments, approximated 10% for the ouabain responsive group and 33.3% for the noise responsive group.

### Protein identification via hydrogel extraction and LC-MS/MS analysis

A third slide containing a tissue section from the control and experimental condition was prepared for protein identification via trypsin-loaded hydrogel extraction. Briefly, hydrated disks of 18% polyacrylamide gel, approximately 3 mm in diameter, were soaked in a 0.1 mg/ml trypsin solution (made in 100 mM ammonium bicarbonate) for 20 min, after which time the excess liquid was removed. After washing as noted above (see wash steps prior to protein imaging), trypsin-loaded hydrogels prepared as previously described (Rizzo et al., 2017) were placed on cochlea tissue sections in the area of the auditory nerve and lateral wall, and then placed in an incubation chamber with 100 mM ammonium bicarbonate and kept at 37°C overnight.

The incubation chamber was removed from the oven and the peptides were extracted by adding 20 μl 60% acetonitrile with 0.1% trifluoroacetic acid and centrifuging at 9,000 rpm for 5 min. The supernatant was collected in a microcentrifuge tube. That process was repeated three more times with 20 μl 100 mM ammonium bicarbonate and one final time with 60 μl 95% acetonitrile with 0.1% trifluoroacetic acid. The supernatant (~180 μl) was dried with a Speedvac and kept at 4°C prior to analysis.

Once the extraction process was completed, the combined supernatants were dried down using a centrifugal vacuum concentrator and reconstituted in 0.1% TFA. Extracts were desalted and purified using C18 ZipTips, and analytes eluted into low-retention microcentrifuge tubes. The samples were again dried and reconstituted in 10 μl of 0.1% formic acid for LC–MS/MS analysis. Resulting peptides were analyzed by a 70 min data dependent LC-MS/MS analysis. Briefly, 4 μl of peptides were auto-sampled onto a 200 mm by 0.1 mm, self-packed analytical column (Jupiter 3 micron, 300A) coupled directly to an LTQ (ThermoFisher, Waltham, MA) using a nano-electrospray source and resolved using an aqueous to organic gradient at a 500 nl/min flow rate. A single full scan mass spectrum followed by 5 data-dependent tandem mass spectra (MS/MS) were collected throughout the run and dynamic exclusion was enabled to minimize acquisition of redundant spectra. MS/MS spectra were searched via SEQUEST against a mouse protein database along with reversed version for each of the entries. Identifications were filtered (FDR set to 5% at peptide and protein level with minimum of 2 peptides per protein) and collated at the protein level using Scaffold (Proteome Software Inc, Portland, OR). Enrichment analysis of biological process terms was conducted with AmiGO (Ashburner et al., 2000; Mi et al., 2017; The Gene Ontology, 2017) using protein accession numbers as input.

### Immunohistochemistry

Frozen tissue sections embedded in Tissue-Tek OCT compound (Electron Microscopy Science, Fort Washington, PA) were permeabilized using 0.2% triton-x 100 followed by incubation in 0.2% bovine serum albumin (BSA). Sections were incubated overnight in primary antibody against Parvalbumin alpha (P3088, Sigma Aldrich) diluted in 0.2% BSA. Sections were washed with 0.2% triton in PBS and then incubated with biotinylated secondary antibody followed by conjugation to Fluorescein Avidin DCS (Vector Labs, Burlingame, CA). Nuclei were counterstained with propidium iodide (PI). Confocal image stacks were acquired using a Zeiss LSM 880 NLO (Carl Zeiss Inc., Jena, DE, Germany) with Zen acquisition software (Zeiss United States, Thornwood, NY). Images were taken at one μm intervals with image sizes of 134.95 μm (x) X 134.95 μm (y). Images were processed using Zen 2012 Blue Edition (Carl Zeiss Microscopy GmbH) and Adobe Photoshop CC (Adobe Systems Incorporated).

## Results

### Auditory function decline is associated with the degree of cochlear insult

**Figure 2:**
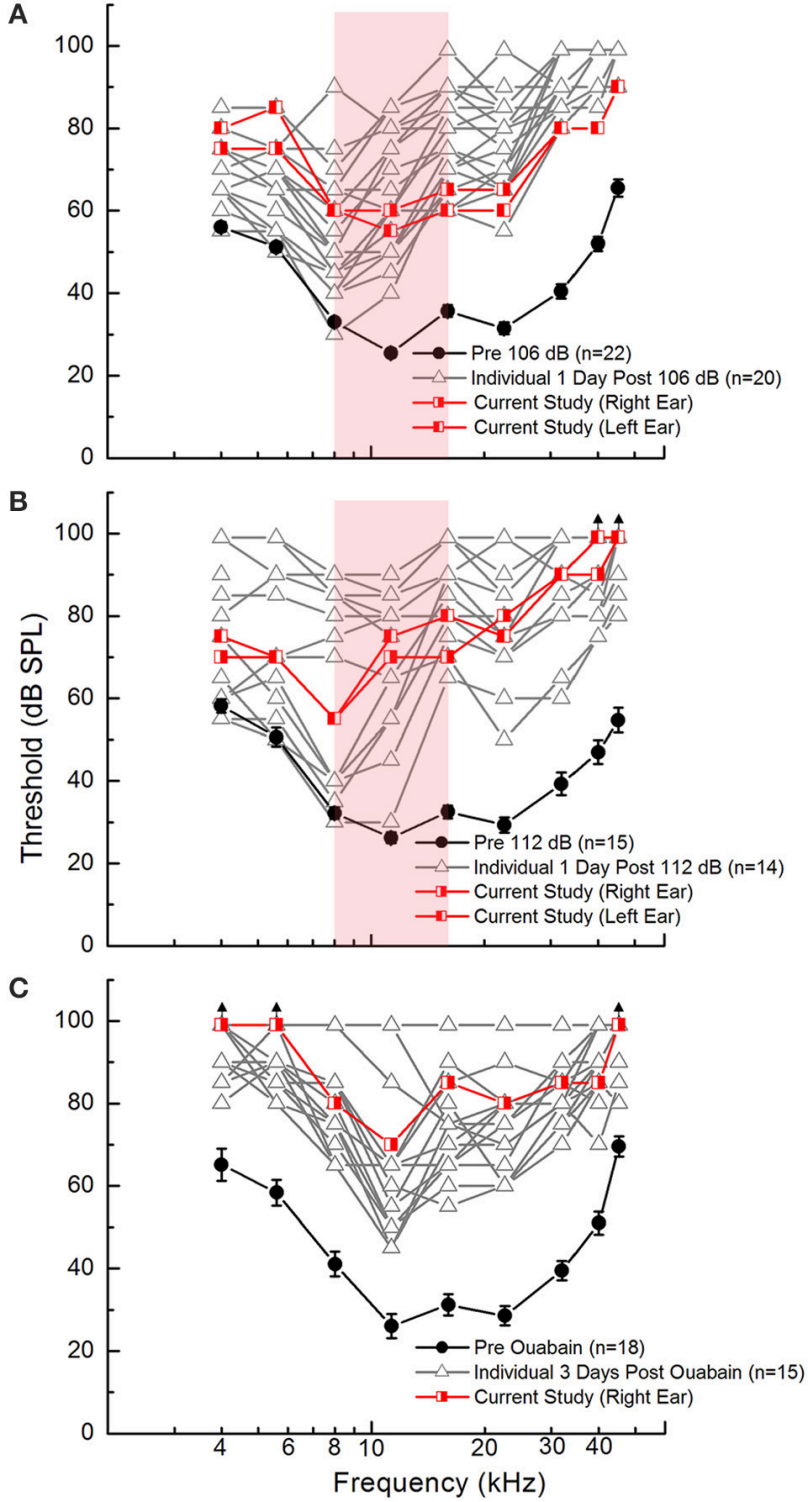
Auditory function declines as a result of cochlear insults in young adult CBA/CaJ mice. ABR Wave I thresholds are elevated 1 day after noise exposure with 106 dB **(A)** or 112 dB **(B)** and 3 days after ouabain (Ouab) administration **(C)** as compared to pre-injury measures. Experimental measures are plotted in red and the pre-injury measures in black. Additional measures collected from similar age animals for each injury model are shown [gray lines; unpublished and re-plotted data from (Panganiban et al., 2018)] to demonstrate that the subjects used in this study exhibit hearing deficiencies representative for each injury model. Red-shaded area in **(A,B)** represents frequency range of octave band noise (8–16 kHz). Error bars represent SEM. Sample sizes (ears per group) are *n* = 22 for 106 dB noise exposure, *n* = 15 for 112 dB noise exposure, *n* = 18 for Ouab administration.

Acoustic overexposure was modeled in mice using octave band noise (8-16 kHz) at sound pressure levels of 106 or 112 dB to induce auditory function decline. Ouabain exposure (Schmiedt et al., 2002), which has been shown to cause dramatic auditory functional decline and cochlear nerve transcriptomic changes (Lang et al., 2011, 2016), was used as a model of injury more severe than either noise exposure. As shown in Figure 2, ABR measurement revealed that the degree of auditory functional decline exhibited by the subjects used in the current study (red lines) were within normal ranges of threshold responses (gray lines) for each injury model. At frequencies 8–16 kHz (Figures 2A,B, shaded area), ABR Wave I thresholds for subjects used in the current study are seen to increase for mice receiving 106 or 112 dB exposure (Figures 2A,B). However, at higher frequencies (>11.3 kHz), the 112 dB exposure results in threshold elevation of an additional 15 dB when compared to that of 106 dB exposure. Figure 2C demonstrates that the ouabain exposure causes more severe auditory functional decline based on ABR wave I threshold responses. At frequencies of 4, 5.6, and 45.2 kHz, there was no measurable response (black arrows) in the ouabain exposed ear (right ear) (Figure 2C).

### Unique m/z signals are localized to injured cochlear tissues

**Figure 3:**
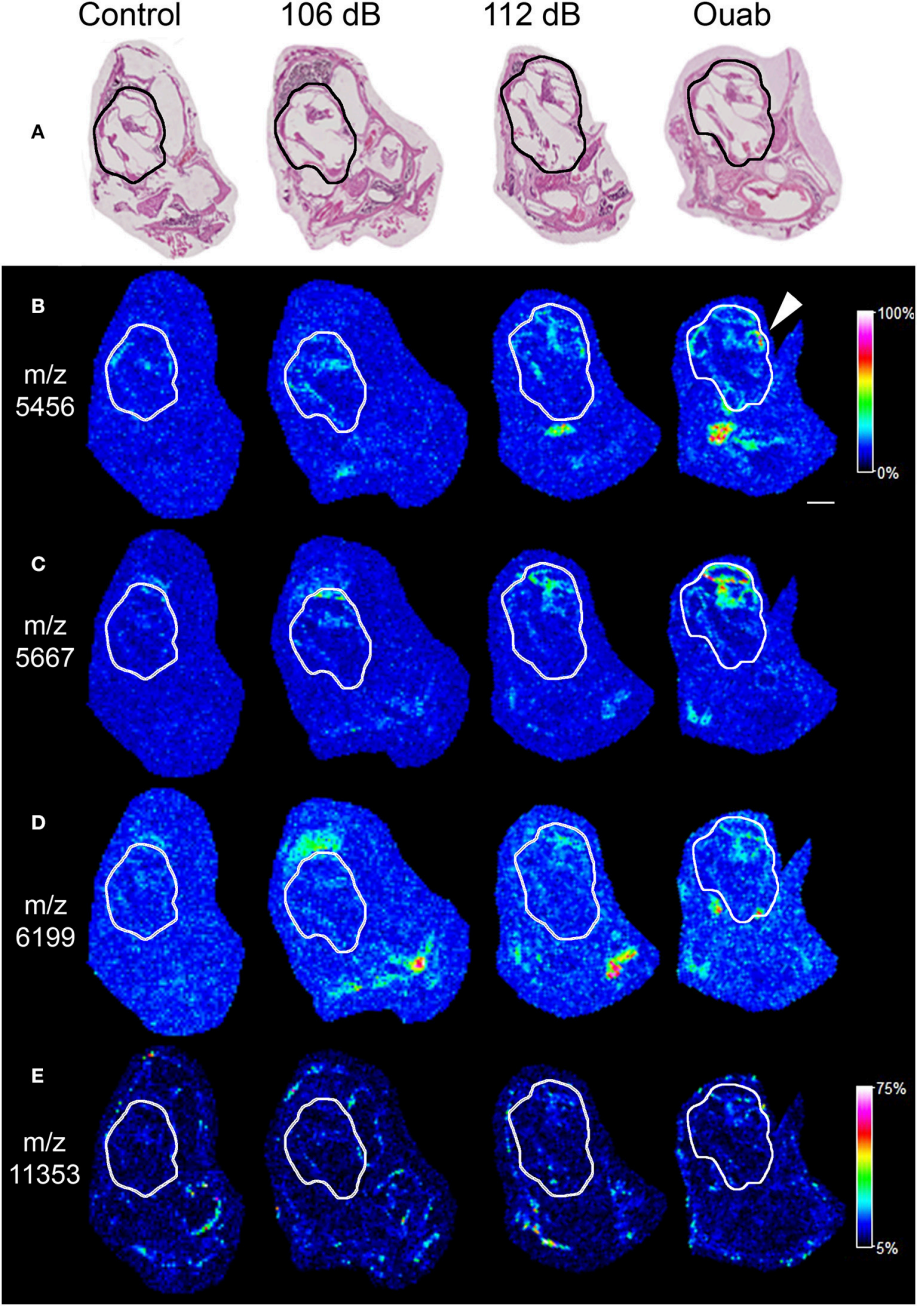
Imaging mass spectrometry of mouse cochlear sections. **(A)** Hematoxylin and eosin stain of cochlear midmodiolar sections following imaging mass spectrometry revealing the areas of the cochlea (outlined). **(B)** The protein signal at m/z 5456 is expressed throughout the cochlea with the highest density seen in the middle portion (arrowhead) of the cochlea exposed with ouabain. The protein signals at m/z 5667 **(C)** and m/z 6199 **(D)** demonstrate high intensity levels in the apical regions of the cochlea exposed with ouabain. **(E)** In contrast, the protein signal at m/z 11353 shows reduced background and localizes to the cochlea. The images of m/z 5456 **(B)**, m/z 5667 **(C)**, m/z 6199 **(D)**, and m/z 11353 **(E)** all demonstrate increasing cochlear protein signals localized to prominent structures with raising insult intensity. Images were generated at 50 μm spatial resolution. Intensity scale in **(B)** applies to **(B–D)**. Scale bar: 500 μm.

To identify the biomolecules responsive to insults, cochleas representing each injury condition were processed for MALDI-TOF IMS and protein identification. Here we used an acidified ethanol fixation technique that has been shown to adequately maintain tissue structures and reduce cross-linking artifacts often seen in formalin-fixed tissues (Grey et al., 2010). Mid-modiolar sections of the cochlea for each injury condition were mounted on conductive slides (one section per test condition totaling four sections per slide) and analyzed on the RapiFlex Tissue Typer. Data resulting from the MALDI-TOF IMS analysis was analyzed to find proteins (m/z signals) that showed precise distribution patterns within sub-regions of the cochlea. Figure 3 shows ion density mapping for four of the m/z signals identified by this analysis generated at 50 μm spatial resolution. Hematoxylin and eosin staining of the sections confirmed that the m/z signals were located within the cochleas (Figure 3A). The protein signal at m/z 5456 (Figure 3B) demonstrates localization throughout all cochlear turns of the ouabain-exposed ear, with the highest density levels corresponding to the middle portion of the cochlea (Figure 3B, arrowhead). The m/z 5456 signal is also seen throughout the cochlea of the 112 dB exposed sample but shows reduced intensity in the 106 dB exposed and control cochleas. Protein signals at m/z 5667 and m/z 6199 (Figures 3C,D, respectively) show high abundance and localization to the apical region of the cochlea for the 112 dB noise and ouabain exposed cochleas. In contrast, the protein signal at m/z 11353 (Figure 3E) is the least abundant in the ouabain sample, but nonetheless demonstrates localization to cochlear regions. Interestingly, all the signals exhibit a cochlear density that increases with the level of insult, suggesting that the proteins represented by these signals are involved in a biological response to trauma.

### Observations of spatially distinct m/z signals in the cochlear middle turn

**Figure 4:**
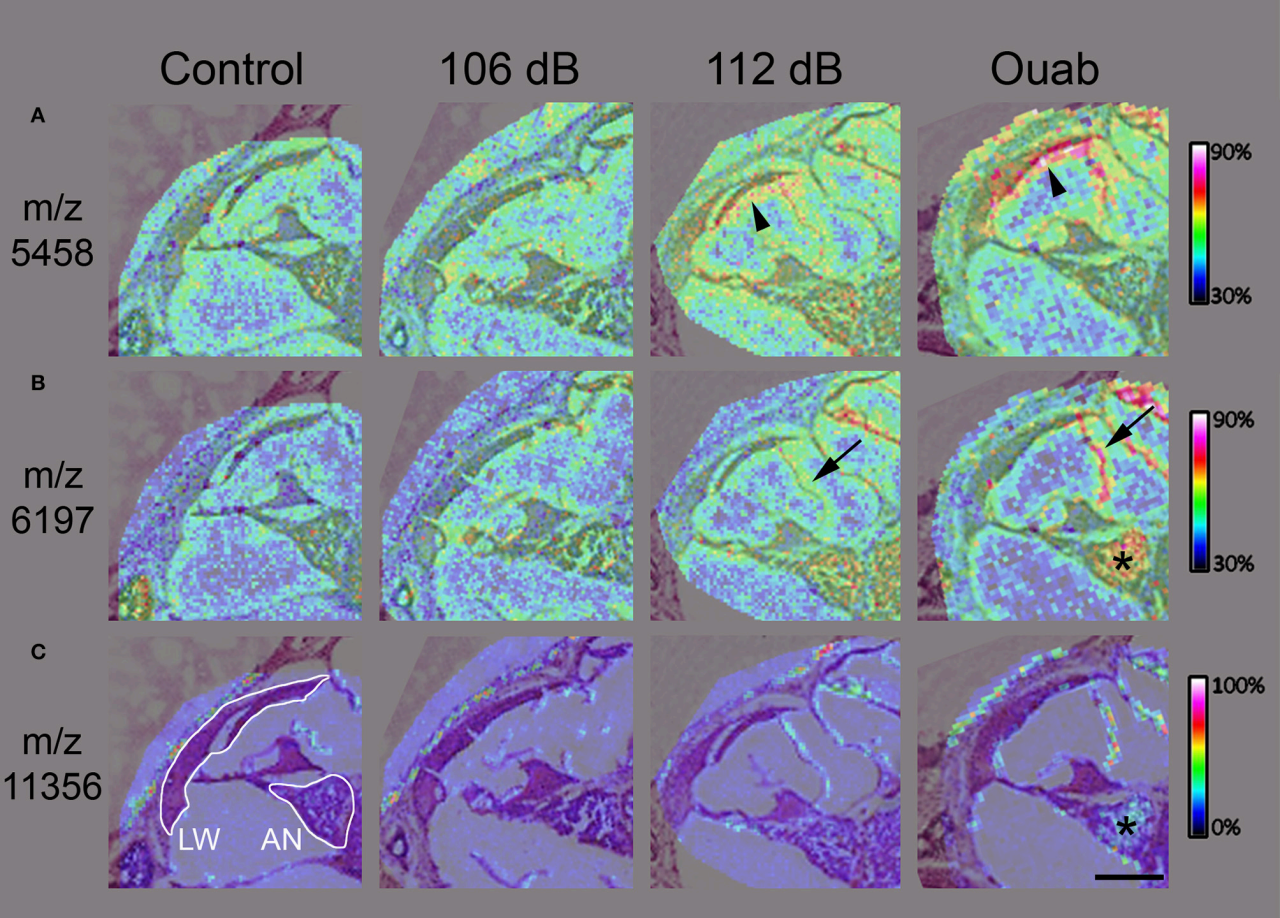
High resolution imaging mass spectrometry in mouse cochlear sections. Two-dimensional ion density map overlay of mass-to-charge ratios 5458 **(A)**, 6197 **(B)**, and 11356 **(C)** in the middle turn of control, 106 or 112 dB noise exposed, and ouabain exposed cochlea. The protein signal at m/z 5458 **(A)** is present in the lateral wall (LW, arrowhead) of the 112 dB ear with even higher density observed in the LW (arrowhead) of the ouabain exposed cochlea, while showing lower signal presence in the control and 106 dB middle turns. Similarly, the signal at m/z 6197 **(B)** displays increasing density corresponding to the severity of the cochlear insult, with localization to auditory nerve (asterisk) and Reisner's membrane (arrow). Interestingly, the m/z 11356 **(C)** demonstrates localization of protein signal in only the ouabain exposed ear. Images were generated at 10–15 μm spatial resolution. Scale bar: 200 μm.

In MALDI TOF IMS the size of the laser focal point determines the resolution of the resulting mass spectrum image for any given tissue section. During data acquisition, a mass spectrum is associated with each focal point, or pixel, and selection of any one mass-to-charge protein signal will show its localization and intensity information across the entire region of interest (Chaurand et al., 2005). For higher resolution of protein signal localization within the cochlea, MALDI IMS analysis was repeated at 10–15 μm spatial resolution for regions corresponding to the cochlear middle turn. The protein signals at m/z 5458 and m/z 6197 were localized to the lateral wall and/or auditory nerve, respectively (Figures 4A,B). These m/z signals again demonstrated increasing signal density with increasing injury level as can be seen in the lateral wall (Figure 4A, arrowhead), Reissner membrane (Figure 4B, arrow), and auditory nerve (Figure 4B, asterisk) of 112 dB and ouabain treated samples. Interestingly, some signals localized only to the auditory nerve for the ouabain exposed cochlea, as exemplified in Figure 4C for signal m/z 11356.

### Tentative identification of m/z signals

**Figure 5:**
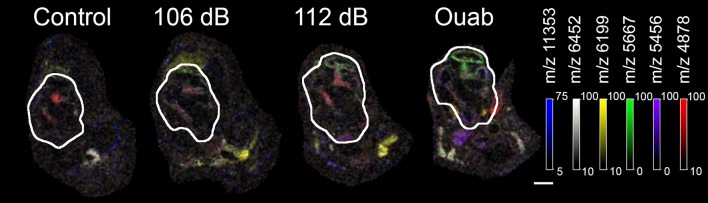
Overlay of selected protein signals. Protein signals at m/z 11353 (blue), m/z 6452 (white), m/z 6199 (yellow), 5667 (green), m/z 5456 (purple), and m/z 4878 (red) were over-layed to show the potential interactions of the candidate proteins in the cochlea (outlined). These six protein signals were selected for further investigation as molecules responsive to cochlear injury. Images were generated at 50 μm spatial resolution. Scale bar: 500 μm.

Six m/z signals that were localized to the auditory nerve and/or lateral wall—m/z 11353, m/z 6452, m/z 6199, m/z 5667, m/z 5456, and m/z 4878—were selected for further investigation (Figure 5). Overlay of these protein signals highlighted that they have distinct spatial localization within the cochlea and that their density was dependent on the severity of injury (Figure 5). To identify the proteins present in the injury samples, a search was performed using the TagIdent tool on ExPaSY (Wilkins et al., 1999) to establish a list of protein candidates for each m/z signal. To account for differences in molecular weight (MW) that might arise due to protein modification(s), searches were done using a MW range of ± 1000 daltons. Results of this analysis are shown in Table 1. For m/z signals 4878, 5456, 5667, 6199, 6452, and 11353, the searches found 146, 124, 134, 138, 150, and 465 candidate proteins, respectively.

**Table 1:** Number of candidate proteins identified by Proteomic Database Query.

**Signal^a^**	**MW range^b^**	**Candidates^c^**
4878	3878–5878 (21%)	146
5456	4456–6456 (18%)	124
5667	4667–6667 (18%)	134
6199	5199–7199 (16%)	138
6452	5452–7452 (15%)	150
11353	10353–12353 (9%)	465

*Lists of proteins within ± 1 kDa of the observed m/z were developed for each protein signal of interest*.

a *50 μm scan m/z signal*.

b *Molecular weight (MW) range for the signal ± 1 kDa; maximum percent change relative to the signal is indicated in parentheses*.

c *Number of candidate proteins matching the MW range*.

### Refinement of protein candidates using mRNA expression data

Considering the cost of protein validation methods (antibody purchasing, time, expensive proteomic techniques) as well as the inability to identify proteins based on mass alone, an alternative means of corroboration was desired. We therefore sought to refine the list of candidates by evaluating pre-existing transcriptomic data sets from microarray analysis of mRNA expression patterns. Although there is not necessarily a direct correspondence between mRNA expression and protein abundance in the unperturbed mammalian system (Vogel and Marcotte, 2012; Kosti et al., 2016), our aim here was to identify injury responsive biomolecules that demonstrate dynamic changes in protein and mRNA expression. The protein densities that increase with level of injury (Figures 4B,C) in MALDI are presumed to correspond to changes in transcriptional activity. Therefore, microarray data generated previously by our group (Lang et al., 2011; Panganiban et al., 2018) for these same two models of cochlear injury were used to refine the lists of candidate proteins, limiting them to genes exhibiting significant differential expression following injury treatment.

Normalized gene expression datasets for auditory nerve tissue either at 3 days post ouabain exposure or 1 day post 106 dB noise exposure generated by our lab were obtained from NCBI Gene Expression Omnibus (accessions GSE59416 and GSE100365) (Lang et al., 2011; Panganiban et al., 2018). Significant differential expression was defined as statistical significance (*p*-value < 0.05) for the injury treatment compared to control; a summary of the comparison findings for candidate proteins is presented in Table 2. In general for any given m/z candidate pool, approximately 5% of the candidates were differentially expressed in response to noise and approximately 15% of the candidates were differentially expressed in response to ouabain. Allowing for mRNAs significantly differentially expressed in either of the injury models, there were 21, 22, 24, 25, 32, and 128 proteins corresponding to m/z signals 4878, m/z 5456, m/z 5667, m/z 6199, m/z 6452, and m/z 11353, respectively, reducing the number of possible candidates by more than 70% for each signal of interest.

**Table 2:** Potential protein identifications.

**Signal^a^**	**Candidates^b^**	**Noise^c^ mRNA response**	**Ouabain^d^ mRNA response**	**mRNA^e^ response**	**List reduction (%)**
4878	146	6 (4%)	16 (11%)	21 (14%)	86
5456	124	4 (3%)	19 (15%)	22 (18%)	82
5667	134	4 (3%)	21 (16%)	24 (18%)	82
6199	138	6 (4%)	22 (16%)	25 (18%)	82
6452	150	8 (5%)	28 (19%)	32 (21%)	79
11353	465	37 (8%)	117 (25%)	128 (28%)	72

*Protein candidate lists developed based on mass (MW ± 1 kDa), were refined via transcriptomic analysis to identify proteins with statistically significant differential expression (DE) (p < 0.05)*.

a *50 μm scan m/z signal*.

b *Number of candidate proteins matching the signal ± 1 kDa*.

c *Number of candidates with differential mRNA expression (p < 0.05) in noise-treated samples*.

d *Number of candidates with differential mRNA expression (p < 0.05) in ouabain-treated samples*.

e *Number of candidates with differential mRNA expression for noise or ouabain treatment*.

### Identification of proteins from cochlear tissue sections

A current challenge for proteomic studies is that protein extraction methods may require homogenization of the entire tissue sample, thereby resulting in a loss of spatial information. In the case of *in situ* protein identification from tissue sections, hydrogel technologies are typically applied to facilitate region-specific protein extractions. Recent advances in the hydrogel extraction methodology (Rizzo et al., 2017) have (1) improved the number of proteins that can be identified, and (2) reduced the diameter of the applied hydrogel to ~260 μm, allowing for the proteomic analysis of smaller biological structures such as the mouse cochlea. Therefore, as a second means of limiting the number of candidate proteins corresponding to m/z signals described above, we conducted trypsin-containing hydrogel extraction on distinct regions of the cochlea for the control and ouabain-exposed cochlea, followed by LC-MS/MS analysis.

A total of 198 proteins within a MW range of 10–3,900 kDa were identified by trypsin-containing hydrogel extraction and LC-MS/MS analysis of control and ouabain-exposed cochlea (Table 3), 30 of which are unique to the ouabain-exposed cochlea and 59 unique to the control sample. Proteins found in both samples included the myosins (−1, −3, −4), neurofilaments (light, medium, and heavy), cochlin, myelin basic protein, and sodium/potassium transporting ATPase alpha-1 and alpha-2 subunits. Ontology analysis of the proteins unique to the ouabain-exposed sample revealed platelet aggregation and cellular component organization or biogenesis to be the most overrepresented biological processes. Proteins identified in the ouabain sample that were linked to both enriched processes included vinculin, alpha- and beta-chain fibrinogen, and Wnt-3a. Total spectral counts were used as a semi-quantitative measure of protein abundance in the samples (Lundgren et al., 2010). The protein with the highest abundance in the ouabain-exposed cochlea was complement C3, followed by thrombospondin-1, and beta-chain fibrinogen. In the PANTHER Pathways analysis fibrinogen is also a significant component of the plasminogen activating cascade that was overrepresented in the ouabain exposed cochlea. Ontology analysis of the proteins unique to the control sample detected overrepresented biological processes of response to activity, actin filament-based process, and regulation of biological quality.

**Table 3:** Proteins identified by hydrogel extraction and LC-MS/MS in control and ouabain exposed ear.

			**Total spectral counts**	
**Identified proteins (198)**	**Accession number**	**Molecular weight (kDa)**	**Control**	**Ouabain**
14-3-3 protein beta/alpha	Q9CQV8	28	3	6
14-3-3 protein epsilon	P62259	29	2	3
14-3-3 protein zeta/delta	P63101	28	6	5
2',3'-cyclic-nucleotide 3'-phosphodiesterase	P16330	47	3	0
3-hydroxyacyl-CoA dehydrogenase type-2	A2AFQ2	28	2	0
40S ribosomal protein S3	P62908	27	1	2
60 kDa heat shock protein, mitochondrial	P63038	61	2	0
78 kDa glucose-regulated protein	P20029	72	3	5
Aconitate hydratase, mitochondrial	Q99KI0	85	5	1
Actin, aortic smooth muscle	P62737	42	20	19
Actin, cytoplasmic 1	P60710	42	23	19
Adenylate kinase isoenzyme 1	Q9R0Y5	22	2	1
ADP/ATP translocase 1	P48962	33	8	1
ADP-ribosylation factor 5	P84084	21	2	0
Aldehyde dehydrogenase, mitochondrial	P47738	57	2	1
Alkaline phosphatase, tissue-nonspecific isozyme	P09242	58	0	2
Alpha-1-antitrypsin 1-1	P07758	46	3	4
Alpha-2-HS-glycoprotein	P29699	37	3	1
Alpha-2-macroglobulin	Q61838	166	1	3
Alpha-actinin-1	Q7TPR4	103	6	2
Alpha-actinin-2	Q9JI91	104	6	0
Alpha-enolase	P17182	47	7	4
Androgen receptor	P19091	98	2	1
Annexin A1	P10107	39	0	2
Annexin A2	P07356	39	2	2
Annexin A5	P48036	36	5	2
Annexin A6	P14824	76	2	1
Apolipoprotein A-I	Q00623	31	2	4
Apolipoprotein E	P08226	36	5	2
Aspartate aminotransferase, cytoplasmic	P05201	46	2	0
Aspartate aminotransferase, mitochondrial	P05202	47	4	1
ATP synthase subunit alpha, mitochondrial	Q03265	60	11	1
ATP synthase subunit b, mitochondrial	Q9CQQ7	29	3	0
ATP synthase subunit beta, mitochondrial	P56480	56	11	5
ATP synthase subunit f, mitochondrial	P56135	10	2	1
Basement membrane-specific heparan sulfate proteoglycan core protein	B1B0C7	469	2	3
Beta-enolase	P21550	47	8	0
Biglycan	P28653	42	3	0
Calmodulin	P62204	17	3	0
Calponin-3	Q9DAW9	36	0	2
Calreticulin	P14211	48	2	0
Calsequestrin-1	O09165	46	4	0
Carbonic anhydrase 1	P13634	28	4	0
Carbonic anhydrase 2	P00920	29	5	0
Carbonic anhydrase 3	P16015	29	3	1
Citrate synthase, mitochondrial	Q9CZU6	52	2	1
Cleavage and polyadenylation-specificity factor subunit 6	H3BJW3	63	0	2
Cochlin	Q62507	60	59	46
Collagen alpha-1(I) chain	P11087	138	23	11
Collagen alpha-1(II) chain	P28481	142	6	2
Collagen alpha-1(V) chain	O88207	184	3	3
Collagen alpha-1(VI) chain	Q04857	108	4	0
Collagen alpha-1(XII) chain	Q60847	340	3	2
Collagen alpha-1(XIV) chain	Q80X19	193	4	0
Collagen alpha-2(I) chain	Q01149	130	11	9
Collagen alpha-2(VI) chain	Q02788	110	3	1
Collagen alpha-4(VI) chain	A2AX52	251	0	2
Complement C3	P01027	186	4	14
Creatine kinase B-type	Q04447	43	4	4
Creatine kinase M-type	P07310	43	14	0
Creatine kinase S-type, mitochondrial	Q6P8J7	47	4	0
Cytochrome b-c1 complex subunit 2, mitochondrial	Q9DB77	48	1	2
Desmoplakin	E9Q557	333	2	1
Destrin	Q9R0P5	19	0	2
Dihydropyrimidinase-related protein 3	Q62188	62	2	0
Dynamin-1	P39053	98	0	2
EH domain-binding protein 1-like protein 1	Q99MS7	185	0	2
Electron transfer flavoprotein subunit alpha, mitochondrial	Q99LC5	35	1	2
Elongation factor 1-alpha 1	P10126	50	6	3
Elongation factor 2	P58252	95	2	0
Endoplasmin	P08113	92	3	0
Eukaryotic initiation factor 4A-I	P60843	46	0	3
Fas-binding factor 1	A2A870	130	0	2
F-box only protein 2	Q80UW2	34	2	1
Ferritin light chain 1	P29391	21	4	0
Fibrinogen beta chain	Q8K0E8	55	0	4
Filamin-A	Q8BTM8	281	4	0
Fragile X mental retardation syndrome-related protein 2	Q9WVR4	74	2	0
Fructose-bisphosphate aldolase A	P05064	39	13	4
Fructose-bisphosphate aldolase C	P05063	39	0	3
Glutathione reductase, mitochondrial	P47791	54	0	2
Glyceraldehyde-3-phosphate dehydrogenase	P16858	36	10	5
Glycogen phosphorylase, brain form	Q8CI94	97	1	2
Guanine nucleotide-binding protein G(i) subunit alpha-2	P08752	40	3	0
Guanine nucleotide-binding protein G(I)/G(S)/G(T) subunit beta-2	P62880	37	3	1
HAUS augmin-like complex subunit 3	Q8QZX2	66	0	2
Heat shock cognate 71 kDa protein	P63017	71	4	4
Heat shock protein HSP 90-beta	P11499	83	4	2
Hemoglobin subunit alpha	P01942	15	27	10
Hemoglobin subunit beta-1	P02088	16	34	14
Hemoglobin subunit beta-2	P02089	16	26	10
Hemopexin	Q91X72	51	0	4
Heterogeneous nuclear ribonucleoprotein U	Q8VEK3	88	1	2
Histone H1.3	P43277	22	3	0
Histone H2A type 2-C	Q64523	14	11	7
Histone H2A.Z	P0C0S6	14	6	0
Histone H2B type 1-F/J/L	P10853	14	6	4
Histone H3.3C	P02301	15	3	3
Histone H4	P62806	11	6	2
Isocitrate dehydrogenase [NAD] subunit gamma 1, mitochondrial	P70404	43	2	0
Lactoylglutathione lyase	Q9CPU0	21	2	0
Laminin subunit alpha-2	Q60675	343	1	2
Laminin subunit gamma-1	F8VQJ3	177	1	2
L-lactate dehydrogenase A chain	P06151	36	3	1
L-lactate dehydrogenase B chain	P16125	37	2	3
Lysine-specific demethylase 6B	Q5NCY0	176	0	2
Malate dehydrogenase, mitochondrial	P08249	36	5	4
MCG13402, isoform CRA_a	Q8BGJ5	57	2	0
MCG140437, isoform CRA_d	G3UW82	223	70	0
Microtubule-associated serine/threonine-protein kinase 4	Q811L6	284	2	0
Mimecan	Q62000	34	5	0
Molybdenum cofactor biosynthesis protein 1	Q5RKZ7	70	1	2
Myelin basic protein (Fragment)	F6RT34	23	9	4
Myelin protein P0	P27573	28	4	5
Myoglobin	P04247	17	6	0
Myosin light chain 1/3, skeletal muscle isoform	P05977	21	11	1
Myosin regulatory light chain 2, skeletal muscle isoform	P97457	19	7	0
Myosin, heavy polypeptide 13, skeletal muscle	B1AR69	224	28	0
Myosin-1	Q5SX40	223	105	5
Myosin-3	P13541	224	35	0
Myosin-4	Q5SX39	223	82	0
Myosin-9	Q8VDD5	226	10	6
Neurofilament heavy polypeptide	P19246	117	5	1
Neurofilament light polypeptide	P08551	62	7	4
Neurofilament medium polypeptide	P08553	96	4	2
Nucleophosmin	Q61937	33	2	0
Parvalbumin alpha	P32848	12	5	1
Peptidyl-prolyl cis-trans isomerase A	P17742	18	2	2
Periaxin	O55103	148	14	4
Periostin	Q62009	93	3	0
Peroxiredoxin-1	P35700	22	2	0
Phosphoglycerate kinase 1	P09411	45	6	4
Phosphoglycerate mutase 2	O70250	29	3	1
Pig Trypsin Precursor	P00761	24	3	1
Plastin-2	Q61233	70	2	0
Polyubiquitin-B	P0CG49	34	2	1
Prelamin-A/C	P48678	74	0	2
Protein Ahnak	E9Q616	604	2	1
Protein Arid1b	E9Q4N6	191	0	2
Protein Col22a1	E9Q7P1	160	9	0
Protein Col6a3	E9PWQ3	354	5	1
Protein disulfide-isomerase A6	Q922R8	48	2	2
Protein Fga	E9PV24	87	0	2
Protein Iqgap3	F8VQ29	185	2	0
Protein NDRG1	Q62433	43	3	1
Protein S100-A9	P31725	13	1	5
Protein Wnt-3a	P27467	39	0	2
Prothrombin	P19221	70	2	0
Putative adenosylhomocysteinase 3	Q68FL4	67	1	2
Pyruvate dehydrogenase E1 component subunit alpha, somatic form, mitochondrial	P35486	43	2	0
Pyruvate kinase isozymes M1/M2	P52480	58	16	2
Ras GTPase-activating-like protein IQGAP1	Q9JKF1	189	2	0
Rho GDP-dissociation inhibitor 1	Q99PT1	23	2	0
RNA-binding protein 42	Q91V81	50	1	3
Ryanodine receptor 2	E9Q401	565	2	0
Sarcalumenin	Q7TQ48	99	2	0
Sarcoplasmic/endoplasmic reticulum calcium ATPase 1	Q8R429	109	27	0
Serine/threonine-protein phosphatase 2A 65 kDa regulatory subunit A alpha isoform	Q76MZ3	65	2	1
Serotransferrin	Q921I1	77	2	6
Serum albumin	P07724	69	8	5
Serum amyloid P-component	P12246	26	0	2
Sodium/potassium-transporting ATPase subunit alpha-1	Q8VDN2	113	8	8
Sodium/potassium-transporting ATPase subunit alpha-2	Q6PIE5	112	5	5
Solute carrier family 12 member 2	P55012	131	2	0
Spectrin alpha chain, non-erythrocytic 1	P16546	285	2	3
Spectrin beta chain, non-erythrocytic 1	Q62261	274	2	3
S-phase kinase-associated protein 1	Q9WTX5	19	3	0
Syntaxin-1B	P61264	33	0	2
Talin-1	P26039	270	3	2
Thrombospondin-1	P35441	130	0	5
Titin	A2ASS6	3906	5	2
TPR and ankyrin repeat-containing protein 1	Q8BV79	343	0	2
Transcription factor HIVEP2	Q3UHF7	267	0	2
Transitional endoplasmic reticulum ATPase	Q01853	89	2	4
Transketolase	P40142	68	2	1
Trifunctional enzyme subunit alpha, mitochondrial	Q8BMS1	83	2	0
Triosephosphate isomerase	P17751	32	3	2
Tubulin alpha-1A chain	P68369	50	10	9
Tubulin beta-4B chain	P68372	50	7	4
Tubulin beta-5 chain	P99024	50	8	4
Tyrosine-protein phosphatase non-receptor type 12	P35831	87	2	0
U3 small nucleolar RNA-associated protein 14 homolog B	Q6EJB6	86	1	2
Ubiquitin-like modifier-activating enzyme 1	Q02053	118	2	0
Uncharacterized protein	E9Q070	34	2	0
Uncharacterized protein	D3Z2H9	29	2	0
Vimentin	P20152	54	4	6
Vinculin	Q64727	117	0	2
Vitamin K-dependent protein Z	Q9CQW3	44	2	0
Vitronectin	P29788	55	5	3
Y-box-binding protein 2	Q9Z2C8	38	2	0

### Synthesis of proteomic and transcriptomic findings

Comparison of the proteins identified by LC-MS/MS analysis with the candidate proteins generated from protein database query for each m/z of interest revealed that there were five proteins in common and these corresponded variously to four of the six m/z signals (Table 4). Complement c3g fragment was tentatively identified to represent the protein signals at m/z 4878, m/z 5456, and m/z 5667. In addition to the complement fragment, thrombin light chain was also found to be a possible match for m/z 4878. Three tentative protein identifications were made for the protein signal at m/z 11353: c3-beta-c, Histone H4, and Parvalbumin alpha. These findings were then evaluated against the refined candidate lists generated following mRNA differential expression analysis. Among the five common proteins, two corresponding mRNAs were found to have significant differential expression in either the noise- or ouabain-injury data sets (Table 4), namely Thrombin light chain and Parvalbumin alpha. Based on these findings, we conclude that m/z signal 4878 corresponds to Thrombin light chain (a component of the enzyme necessary for the conversion of fibrinogen to fibrin) and that signal m/z 11353 corresponds to Parvalbumin alpha, a protein involved in the regulation of calcium ion concentration within the cellular cytoplasm.

**Table 4:** LC-MS/MS Validation of protein candidates satisfying MW ± 1 kDa criteria.

**Signal^a^**	**Accession**	**Protein**	**Chain**	**MW (Da)**	**Probe set**	***p*-value**
4878 5456 5667	P01027	Complement C3g fragment	955–1001	4950	N/A	N/A
4878	**P19221**	**Prothrombin (Thrombin Light Chain)**	**325–360**	**4047**	**1418897_at**	**0.045**
11353	P01027	C3-beta-c	569–666	10470	N/A	N/A
	P62806	Histone H4	2–103	11236	N/A	N/A
	**P32848**	**Parvalbumin alpha**	**2–110**	**11799**	**1417653_at**	**0.024**

a *50 μm scan m/z signal*.

*Candidates in bold satisfied the combinatorial analysis criteria (MW ± 1 kDa) and differential expression (p < 0.05)*.

**Figure 6:**
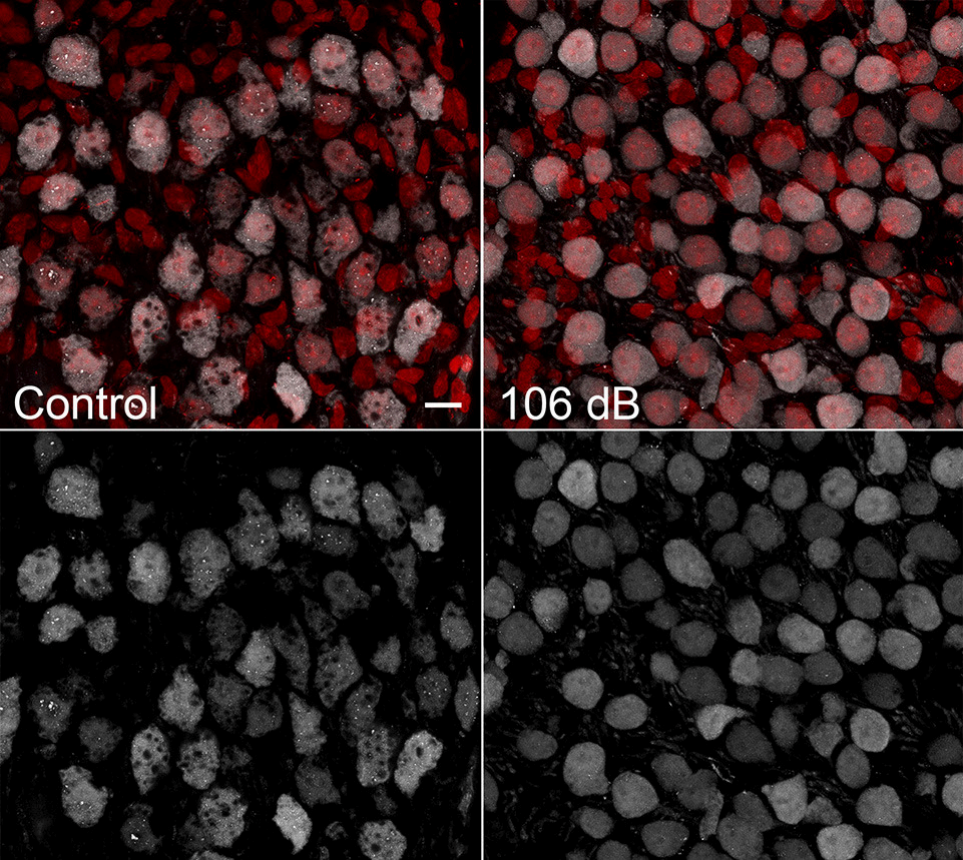
Validation of m/z 11353 protein candidate Parvalbumin alpha. Immunostaining for Parvalbumin alpha in CBA/CaJ cochlear frozen sections detects Parvalbumin alpha in a subset of auditory nerve neuronal cells. One day after noise exposure at 106 dB, Parvalbumin alpha distribution was somewhat altered, with immunoreactivity distributed more uniformly throughout the cytoplasm. Scale bar: 10 μm.

A validation experiment was performed for the protein Parvalbumin alpha to confirm protein localization in the auditory nerve as indicated by the imaging results for signal m/z 11353. As shown in Figure 6, Parvalbumin alpha was detected in spiral ganglion neurons in unexposed cochlea. One day post noise exposure at 106 dB, Parvalbumin alpha distribution appears more diffuse within the cellular cytoplasm. These results agree with previous studies of parvalbumin expression (Soto-Prior et al., 1995).

## Discussion

Here we demonstrate an analytical approach for identifying proteins that exhibit distinct spatial and regulatory behaviors in tissue in response to injury. Our method involves MALDI-TOF IMS visualization of fixed tissue sections and resolution of MALDI-TOF IMS findings with transcriptomic analysis and hydrogel extraction coupled with LC-MS/MS analysis. The appeal of the MALDI-TOF IMS analysis is that it does not require any a priori knowledge of protein sequence or antibodies. Moreover, the analysis requires only a minimal amount of tissue, here performed on 10 μm tissue sections. Although the initial MALDI-TOF IMS results are limited in terms of protein identity, we show that this ambiguity can be dramatically reduced using the accompanying transcriptomic and LC-MS/MS analyses, leading to high probability identifications of the proteins of interest.

MALDI TOF IMS is a revolutionary analytical tool that can be used to understand protein spatial distribution within tissue, as demonstrated here in the context of cochlear response to injury. Previous imaging mass spectrometry analysis of cochlear tissue sections was able to localize phosphatidyl choline species to distinct cochlear regions (Takizawa et al., 2011). However, that analysis was hampered by the use of unfixed cochlear tissues, which resulted in diminished integrity of cochlear structures. Here we show that MALDI TOF IMS analysis may also be performed for unbiased visualization of proteins in fixed cochlear tissue sections. Acid alcohol fixation was vital to preserve fine structures, such as Reissner's membrane, in order to recover intact protein spatial information. The sample preparation method is amenable for visualization at various resolutions (50–10 μm) and for the extraction of proteins by trypsin-containing hydrogel digestion.

To our knowledge, this is the first time that MALDI TOF IMS has been used to discover proteins responsive to cochlear injury. Noise exposure levels of 106 and 112 dB have been documented to induce numerous pathological changes in vital cochlear structures, including hair cells (Chen and Fechter, 2003; Hill et al., 2016), synapse ribbons (Kujawa and Liberman, 2009), auditory nerve (Eskiizmir et al., 2011), and non-sensory cells in the cochlear lateral wall (Hirose and Liberman, 2003). Ouabain exposure has resulted in irreversible pathological alterations of the auditory nerve that contribute greatly to declines in auditory function (Lang et al., 2011; Yuan et al., 2014). Auditory function decline of the noise- and ouabain-exposed subjects observed in this study agree with those of previous studies (Wang et al., 2002; Panganiban et al., 2018). Moreover, the graded level of hearing impairment caused by the low and high noise exposures and the ouabain treatment support the conclusion that these treatments represent a range of injury severity, ranking low to high, respectively. The m/z signals selected for characterization demonstrated a stimulus-dependent expression pattern, with signal density increasing with injury severity. Ion density mapping performed at 50 μm resolution demonstrated that the different signals occupied unique spatial domains within the cochlea. Higher resolution analysis at 10–15 μm provided a more precise assessment of these domains that variously included cochlear substructures such as the lateral wall, auditory nerve, and Reisner's membrane.

This is also the first report of protein identification in cochlear tissue sections by hydrogel extraction coupled with LC-MS/MS. Serial sections from the control and ouabain treated cochlea were used to identify proteins present in the sample by on-tissue hydrogel mediated protein digestion and extraction followed by LC-MS/MS analysis. The detected proteins are known to be associated with vital cochlear structures including the auditory nerve (neurofilaments, myelin basic protein), lateral wall (Na-K ATPase), and hair cells (annexins). Our analysis also distinguished proteins that were present only in the ouabain exposure model, suggesting the role of these proteins in auditory nerve degeneration or repair. These proteins included complement C3, a central molecule in the alternative and classical complement pathway of the immune system, and thrombospondin, a protein involved in hematopoietic stem cell homing and differentiation. This finding is supported by our previous study (Lang et al., 2016) that identified thrombospondin and the complement component 3a receptor 1 (C3ar1) as significantly regulated in the auditory nerve at 3 days post ouabain exposure.

Despite the benefits of the MALDI TOF IMS analysis, the results are limited by the absence of definitive protein identification. Here we helped resolve that ambiguity by incorporating existing transcriptomic data. Refining the protein candidate pool by requiring accompanying mRNA expression change effectively reduced the protein candidate pools by >70%. The potential drawback to imposing differential mRNA expression is that it limits discovery to proteins whose abundance is primarily linked to their transcription level, and thus proteins whose abundance is affected predominantly by post-transcriptional events would be excluded. Nevertheless, the proliferation of transcriptomic studies and the availability of these data through public databases (e.g., Gene Expression Omnibus, ArrayExpress) suggest the possibility that well-suited mRNA expression data can be obtained freely from data repositories, making this combinatorial approach attractive and cost-effective. Despite the efficacy of our approach, components of this methodology may be improved. The MALDI TOF IMS results displayed robust non-specific signaling that may result from the embedding medium or the decalcification method. Additionally, it should be noted that the size restriction applied in this study (signal size ± 1 kDa) imposes a limit that would exclude proteins having larger modifications, such as poly-ubiquitination and glycosylation events. Optimization of tissue preparation and imaging analysis steps may also help limit the non-specific signal.

Here we demonstrate an approach for the molecular imaging and identification of proteins responsive to injury in the mouse adult cochlea. In response to acoustic overexposure or ototoxic drug exposure, proteins were identified that displayed dynamic changes in abundance and distribution in the cochlear lateral wall and auditory nerve. Ambiguities in primary MALDI TOF IMS findings were resolved dramatically by incorporating transcriptomic data and downstream hydrogel digestion/extraction coupled with LC-MS/MS, leading to high probability identification of proteins of interest. This method is applied on fixed tissue specimens, which ensures that morphological structures are well preserved. Thus, it may be useful for analyzing warehoused tissue specimens (Lemaire et al., 2007), including human temporal bone tissues.

## Author contributions

KN, MR, JB, KS, EK, and HL: designed research; KN, MT, and HM: performed research and analyzed data; KN, MR, HL, and JB: wrote the paper.

### Conflict of interest statement

The authors declare that the research was conducted in the absence of any commercial or financial relationships that could be construed as a potential conflict of interest.
